# Temperature Mapping of 3D Printed Polymer Plates: Experimental and Numerical Study

**DOI:** 10.3390/s17030456

**Published:** 2017-02-24

**Authors:** Charoula Kousiatza, Nikoleta Chatzidai, Dimitris Karalekas

**Affiliations:** Laboratory of Advanced Manufacturing Technologies and Testing, University of Piraeus, Karaoli and Dimitriou 80, 185 34 Piraeus, Greece; chkousiatza@unipi.gr (C.K.); nchatzi@unipi.gr (N.C.)

**Keywords:** fused deposition modeling, real-time monitoring, temperature mapping, FEM simulation, thin plates

## Abstract

In Fused Deposition Modeling (FDM), which is a common thermoplastic Additive Manufacturing (AM) method, the polymer model material that is in the form of a flexible filament is heated above its glass transition temperature (T_g_) to a semi-molten state in the head’s liquefier. The heated material is extruded in a rastering configuration onto the building platform where it rapidly cools and solidifies with the adjoining material. The heating and rapid cooling cycles of the work materials exhibited during the FDM process provoke non-uniform thermal gradients and cause stress build-up that consequently result in part distortions, dimensional inaccuracy and even possible part fabrication failure. Within the purpose of optimizing the FDM technique by eliminating the presence of such undesirable effects, real-time monitoring is essential for the evaluation and control of the final parts’ quality. The present work investigates the temperature distributions developed during the FDM building process of multilayered thin plates and on this basis a numerical study is also presented. The recordings of temperature changes were achieved by embedding temperature measuring sensors at various locations into the middle-plane of the printed structures. The experimental results, mapping the temperature variations within the samples, were compared to the corresponding ones obtained by finite element modeling, exhibiting good correlation.

## 1. Introduction

Additive Manufacturing (AM) technologies have been rapidly evolved over the last decades, providing the potentiality of building functional components with complex geometrical features as end-use parts in addition to prototyping purposes. AM is defined by both the International Organization for Standardization and the American Society for Testing and Materials (ISO/ASTM 52900-15) as a “process of joining materials to make parts from 3D model data, usually layer upon layer, as opposed to subtractive manufacturing and formative manufacturing methodologies” [1]. Nowadays, AM processes have established a new industrial revolution [2,3] due to considerable benefits, which they provide compared to the traditional subtractive methods. The most important benefits are: tailored mechanical properties, customized products, many combinations of materials, fast manufacturing and cost-effectiveness.

“Material extrusion” according to ISO/ASTM 52900-15 [1] or Stratasys Fused Deposition Modeling (FDM) is the most widely spread and commonly used process included in the category of AM technologies that utilizes the idea of melting extrusion and resolidification of polymer materials. This technology is employed to fabricate three-dimensional (3D) physical objects directly from computer aided design (CAD) files in a layerwise manner. The FDM method uses thermoplastic as feedstock material to fabricate the structures, such as the commercial Acrylonitrile Butadiene Styrene (ABS) [4,5,6] and the Polylactic Acid (PLA) [7]. During the fabrication process, the model material, which is in the form of a flexible filament, is heated above its glass transition temperature (T_g_) to a semi-molten state in the head’s liquefier. Subsequently, it is extruded in the form of a raster, through the 3D printer’s nozzle, onto the building platform where it rapidly cools, solidifies and bonds with the surrounding material. The formation of bonds among individual roads of the same layer and neighboring layers is facilitated by the diffusion bonding mechanism driven by the thermal energy of the extruded heated material [8]. The end FDM structures are consisted of vertically stacked layers. In addition, each layer is constituted of partially bonded rasters, which show property variations along and across the direction of the roads (rasters). As a result, the end fabricated components can be considered as fibrous composite laminates exhibiting anisotropic properties [9,10].

In the FDM method, the temperature fluctuations developed during the building process play a significant role as they can strongly affect the final quality and mechanical properties of the fabricated part. More specifically, the heating and rapid cooling cycles, which recurrently occur during layer deposition of the filament, are responsible for the generation of non-uniform thermal gradients and the consecutive phase change of the extruded material. Consequently, there is an accumulation of thermal residual stresses and strains induced within the constructed component. When these stress and strain fields are accumulated to a certain level, they can affect the dimensional accuracy of the printed structure as well as cause severe quality issues such as distortion, warpage and even possible part fabrication failure in the form of inter- or/and intra-layer delamination or cracking [11,12]. Furthermore, the temperature history of the FDM constructed parts is very important for understanding the bond formation quality between adjoining rasters and, thus, the eventual mechanical properties of the built structures [13,14,15].

In the scope of optimizing the FDM technique by eliminating the presence of such undesirable effects as mentioned above, real-time monitoring of temperature gradients developed during the fabrication process is considered to be essential for quality control purposes. Nevertheless, in terms of experimental investigation, the demanding printing environment, in which the integrated in situ sensing tools should respond, makes the whole attempt very challenging for researchers. As regards the analytical aspect, finite element modeling is required to simulate the experimental data. At present, the lack of relevant studies necessitates the undertaking of such research activities important in this area of interest. Recently, a methodology has been presented by Kousiatza et al. [16] for monitoring the temperature profiles generated during the building process of rectangular cross section thick specimens. The experimental results of that study indicate that the in-process induced temperature fluctuations influence the magnitude of the measured residual strains.

The objective of the undertaken work was to investigate in situ and in real-time the temperature profiles development within multilayered square thin plates fabricated using the FDM technique. Thin plate configurations are extensively used in many fields of engineering [17]. Nonetheless, these geometries are characterized by the presence of undesirable distortion and warpage phenomena. For that reason, a combined experimental and computational approach was conducted to better understand the temperature evolution during the printing process of such plates. In the presented research work, the recording of temperature changes was achieved by embedding temperature measuring sensors at various locations along and across the whole middle-plane of the printed plate specimens. Furthermore, the experimental results mapping the temperature variations within the samples were compared to the corresponding ones obtained by Finite Element Analysis (FEA).

## 2. Materials and Methods

### 2.1. Experimental Procedure

**Figure 1:**
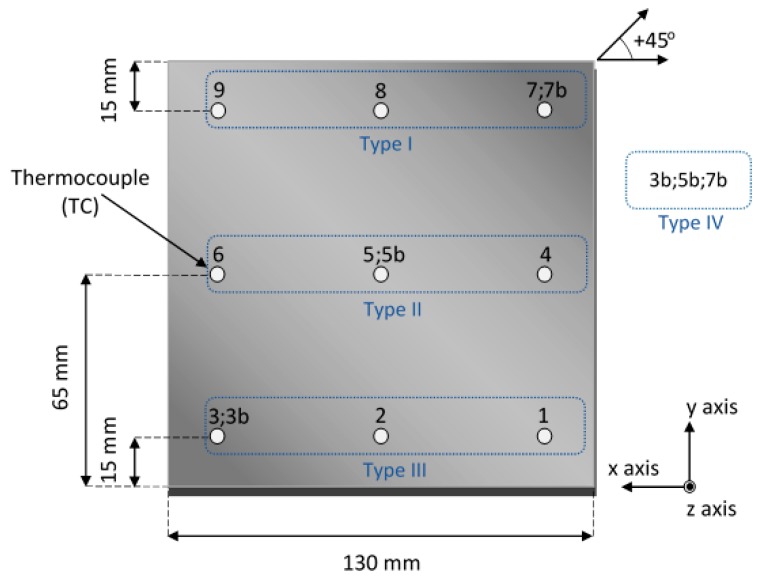
Schematic representation of thermocouples’ locations within the middle-plane of the square plate specimen.

Square thin plate specimens were fabricated out of commercial ABS material on a Dimension Elite (Stratasys, Inc., Eden Prairie, MN, USA) FDM 3D printer. The built test samples had dimensions of 130 mm × 130 mm (width × length) and consisted of 13 layers (3.302 mm of height). Four different types of plate specimens were fabricated for determining in situ and in real-time the temperature variations exhibited during the deposition of the model material. Furthermore, two experimental runs were carried out per each type of sample. In order to record the generated temperature variations during the building process, thermocouple sensors were embedded in various positions within the middle-plane (7th layer) of the FDM multilayered plates. Their locations are presented in Figure 1. In the first type of the conducted experiments, positions 1, 2 and 3 were selected for the embedment of the sensors, while, in the second one, the respective locations were positions 4, 5 and 6. In the third type of plate specimens, the measurements were obtained from the sensors incorporated in locations 7, 8 and 9, whereas, for the fourth type, the thermocouple sensors were placed in a diagonal direction (+45° orientation), and more specifically at positions 7b, 5b and 3b for reasons of comparison. It is noted that the numbering of the integration positions is presented from the right side of the plate samples to the left one, so as to conform to the pattern of the printing direction. The corners of the plate specimen close to the embedding locations 1 and 7 are the places from where the building process of each layer is alternatively initiated (±45°). It is considered that such an array of incorporated thermocouples enables real-time mapping of the developed temperature profiles at the whole samples’ midplanes as well as at the subsequently deposited layers.

For all specimen types, the processing parameters were as follows: the material’s melting temperature within the liquefier was at 270 °C, the envelope temperature environment of the building chamber was automatically controlled by the software at 75 °C, the raster orientation was set to be ±45° representing the machine’s default tool path orientation and the layer thickness of each test sample was chosen to be 0.254 mm. The sensors used to carry out these experiments were K-type thermocouples (±1.5 °C) with a sensing tip of 0.25 mm thickness, while the temperature data recordings were obtained and analyzed through a data acquisition instrument.

**Figure 2:**
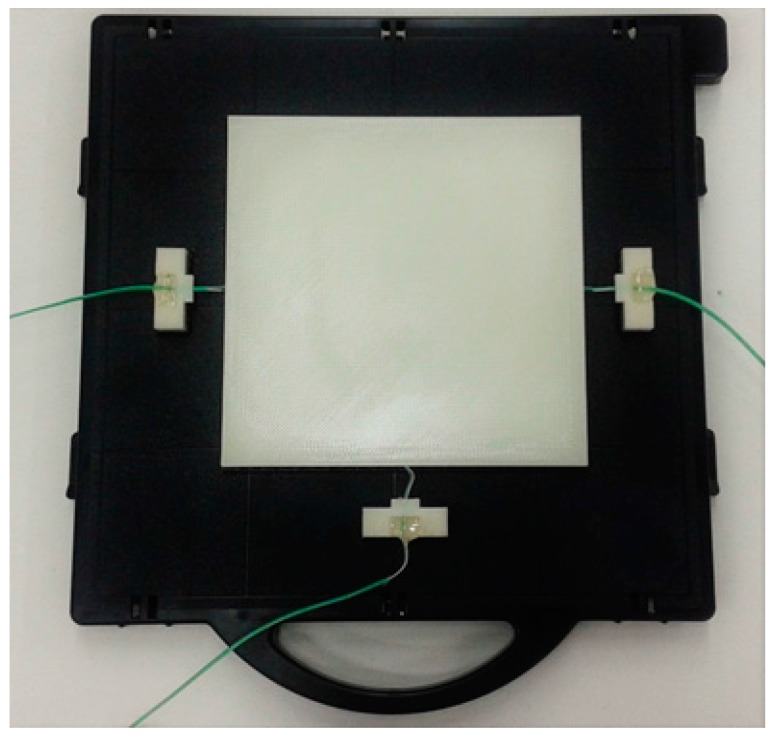
A representative plate specimen simultaneously built with its “alignment holders”.

The building process of the FDM plate specimens started with the deposition of a predefined number of supporting layers. When the last layer of the support material was completed, the layer by layer deposition of the ABS material was enabled until the structure reached the plane at which the thermocouples were to be integrated. At that instant, the FDM 3D printer was paused manually and the printer’s chamber was opened for the implementation of sensors on the surface of the 6th layer. It is mentioned that some specific “alignment holders” were custom designed and simultaneously built with the plate specimen to assure the integration of the sensors at the desired positions within the midplane (see Figure 2). Additionally, embedment grooves (equal to the sensing tip’s diameter) were created in the 6th layer to avoid the contact between the sensors and the nozzle. Afterwards, the printing process continued and the nozzle returned to the location where the layer deposition procedure had stopped in order to deposit the rasters of the next layer. When the 3D printer’s nozzle started building the perimeter of the 7th layer, continuous recording of temperature values was initiated and continued until the completion of the fabrication process.

### 2.2. Finite Element Modeling

In order to simulate the thermal diffusion problem in square thin plate specimens during the FDM building process, the ABAQUS^®^ software was used (ABAQUS, Hibbitt, Karlsson & Sorensen, Inc., Providence, RI, USA). Initially, a square model of 130 mm × 130 mm × 1.778 mm (width × length × height) was designed. This model represents the experimental test sample from the first deposited layer until the plane at which the temperature sensors were integrated (7th layer). Subsequently, in order to simulate the next ABS deposited layer, a new model was designed, having a height of 0.254 mm, on top of the previous one. The new model was meshed and solved using the equations and the boundary conditions that are presented further down. The derived numerical results correspond to the temperature values developed within the 7th layer at the thermocouple locations. The previously described procedure was repeated for every new deposited layer of the experiment until the 13th layer and, separately, for each thermocouple sensor requiring a total of 54 runs.

The governing equation for the thermal analysis is given by:

(1) $$\frac{\partial \left(\rho C_{p}T\right)}{\partial t}=\nabla \cdot k\nabla T+q,$$

where *T* is the temperature, *ρ* the density, *C_p_* the specific heat, *k* the thermal conductivity and *q* the heat generation rate.

The boundary conditions of the outer surfaces of the square specimen are set to be convection:

(2) $$Q=h\left(T-T_{env}\right),$$

where *h* is the heat convection coefficient and *T_env_* the envelope temperature of the chamber (75 °C).

The temperature at the bottom surface of the plate sample being in contact with the platform is set to be constant and equal to the temperature of the platform:

(3) $$T=T^{*}.$$

Since the platform is not self-heating, the temperature was set equal to the envelope temperature of the chamber (75 °C).

For the upper plate’s surface, a known temperature profile based on the experimental data is used as the boundary condition. As mentioned above, for each type of experiments, a set of three sensors were embedded simultaneously within the plate sample at specific locations (15 mm, 65 mm and 115 mm from the right edge). As the printer’s nozzle passed over a particular thermocouple of its corresponding set, a peak value was recorded while the other two sensors measured lower temperatures. The temperature values obtained by the different set of sensors, each time, were determined to be the plate’s upper boundary condition. It is also noted that the FE model was considered as a solid structure. The crisscross raster orientation and the formation of a mesostructure was not taken into account.

The thermal properties of the ABS material used for the simulations are presented in Table 1 and were taken from Sun et al. [15].

In relation to the heat convection coefficient, *h*, considered in the FE analysis, a wide range of values is reported in the literature. According to Li et al. [18], the upper limit of *h* in the FDM process is approximately 140 W/m^2^·K. Bellehumeur et al. [19] refer a range of 50–100 W/m^2^·K for *h*, while Monzón et al. [14] found that a value of 7 W/m^2^·K brings the theoretical solution more closely to their experimental results. In order to examine the influence of *h* at the present thermal diffusion problem, the simulations were repeated for the following three different values: 7 W/m^2^·K, 75 W/m^2^·K and 140 W/m^2^·K as well as for two different thermocouple locations (TC1 and TC7). It was found that, due to the considerable length of the plate, there was no influence of *h* at the points of interest (15 mm, 65 mm, 115 mm). The heat convection coefficient affects the solution only at locations having a distance of 6.6 mm from the plate’s edges. As a result, a mean value of *h* = 75 W/m^2^·K [19,20] was considered in the present simulations.

## 3. Results and Discussion

### 3.1. Comparison of In Situ Monitored and Numerically Derived Temperature Peak Values

**Figure 3:**
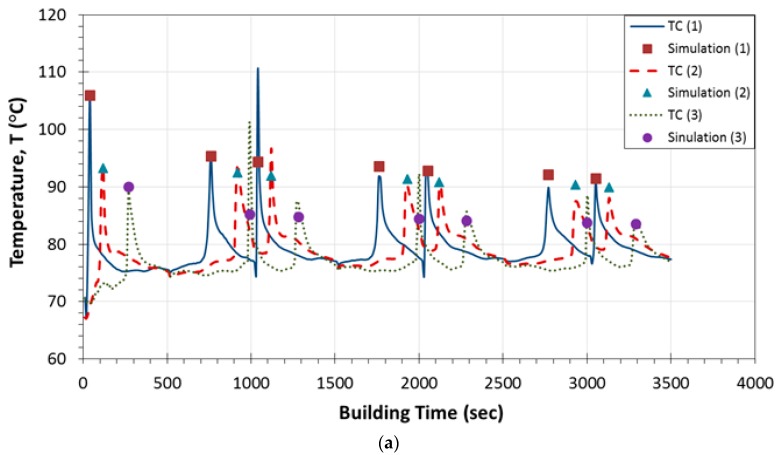

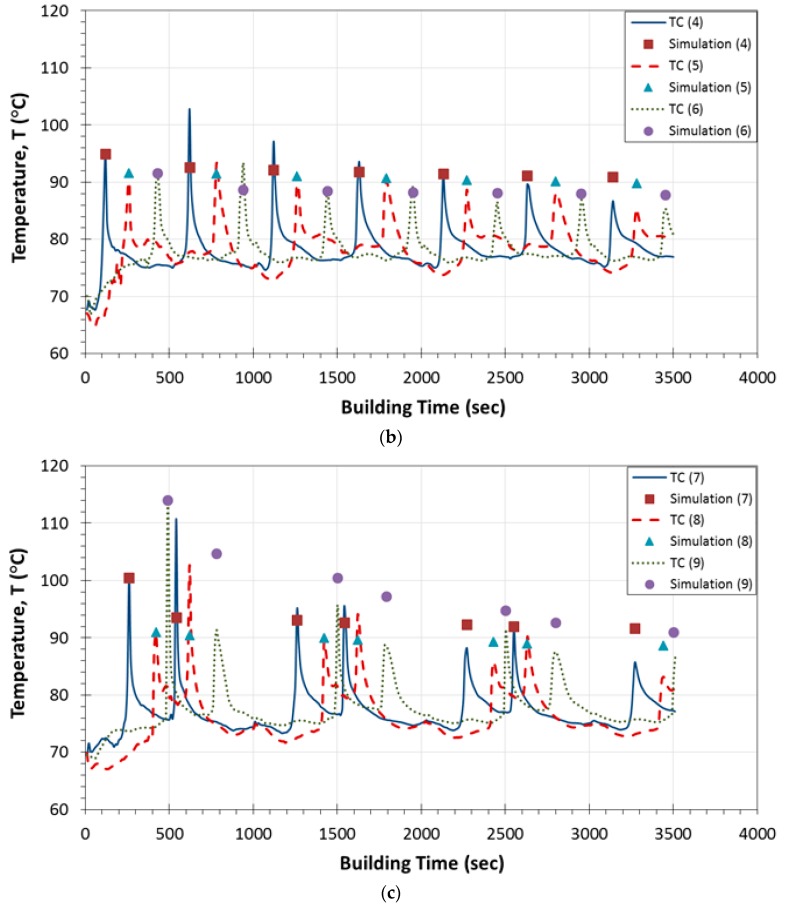
Temperature profiles as recorded by thermocouples in addition to temperature peak values as calculated by FEA for embedding locations: (**a**) 1, 2 and 3; (**b**) 4, 5 and 6; (**c**) 7, 8 and 9.

In Figure 3a–c, the obtained temperature profiles are presented as a function of building time for all thermocouples located in positions 1–3, 4–6 and 7–9, respectively. In each of these diagrams, the temperature peak values are plotted together with the corresponding ones calculated by finite element analysis. The curves in graphs represent the real-time monitoring of temperature variations that take place during the FDM fabrication process, whereas the different types of used symbols correspond to the temperature values derived by the simulations. As it is observed in Figure 3a–c, during the continuous real-time monitoring process, the temperature reaches a peak value prior to a rapid decrease. This peak indicates the passage of the printer’s nozzle exactly above the corresponding thermocouple. In addition, the building process until the completion of each layer has a duration of 500 s while, at this time period, three peak temperature values are observed. Furthermore, the interval time between the appearances of the maximum temperatures differentiates from one diagram to the other (Figure 3a–c) because it is directly related to both the nozzle’s tool path orientation as well as to the sensors’ position within the embedding layer. As regards the specimens where the thermocouples were placed at locations close to the plates’ edges (Figure 3a,c), the recorded temperature profiles exhibited steep fluctuations compared to the respective ones obtained by sensors embedded in locations 4, 5 and 6 (Figure 3b). It is noted that, in Figure 3a,c, the steep peak values are occurring during material deposition of the initial three and two layers (until 1500 s and 1000 s), respectively, while afterwards a declining pattern is recorded from all the temperature measuring sensors. As mentioned in Section 2.2, the recorded measurements at the 7th layer were considered to be the upper boundary condition for the simulations of the plate model. Keeping this assumption in mind, in Figure 3a, a good agreement is demonstrated, in general, between the measured and the simulation based calculated peak values. These temperatures present a difference of 1–5 °C in relation to the corresponding data collected from the thermocouple sensors. Nevertheless, the temperature fluctuations occurred at 1000 s and 2000 s of the printing process at locations 1 and 3 as well as at location 3, respectively, result in much higher deviations between the experimentally and numerically obtained temperature results. In Figure 3c, the significant temperature variations at locations 7, 8 and 9 displayed during the filament deposition of the first two layers are attributed to the nature of the building process. For most of the subsequently built layers, the deviations observed between the recorded temperature peak values and the calculated ones are due to the fact that the formation of a mesostructure was not taken into account at the finite element analysis. In contrast, in Figure 3b, the temperature measurements obtained by sensors located at positions 4, 5 and 6 display uniform temperature profiles during the layerwise deposition of the model filament until the plate’s completion, while a smooth decreasing trend is monitored after approximately 1000 s. For this type of test samples, the numerical and experimental data present the best obtained correlation exhibiting small differences of 3–4 °C. It must be mentioned that, in all cases, the calculated temperature peak values exhibit a decreasing tendency during the layerwise printing process, which arises from the assumptions made for the finite element modeling. For all studied locations, this declining trend reaches a temperature plateau approximately after the completion of the 8th layer except for the case of position 9. Furthermore, in Table 2, the temperature peak values as recorded during the two experimental runs are presented for all the sensors. The maximum difference for all the recorded temperature peak values is calculated to be 3.9 °C.

**Figure 4:**
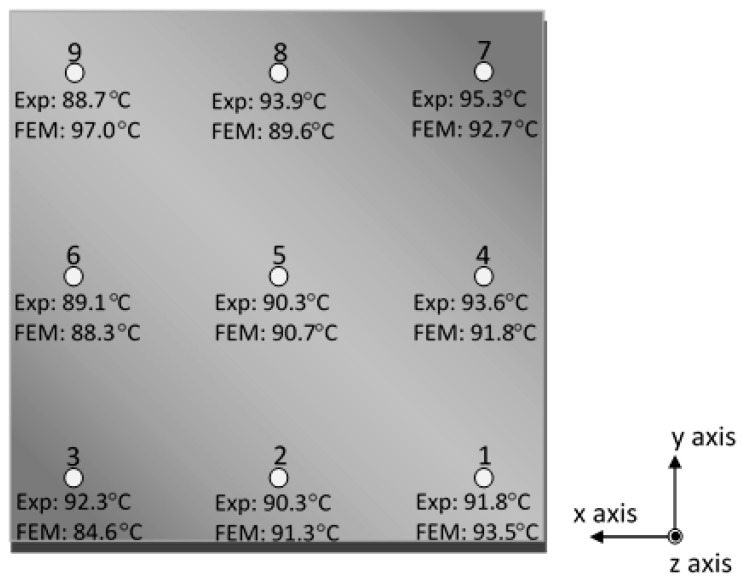
Experimentally obtained temperature values in comparison with numerically calculated ones throughout the 10th layer’s surface (building time: 1500–2000 s).

A comparison between temperature values as measured in real-time throughout the 10th layer’s surface by the thermocouple sensors and as calculated by the finite element model is schematically presented in Figure 4, for the better understanding of the resulted deviations. In general, a good agreement is demonstrated between the experimentally obtained and the numerically calculated temperatures at almost all the embedding locations except for positions 3 and 9. Small differences of 0.4 °C to 1.5 °C can be considered as negligible since they fall within the inherent sensitivity range of the recording sensors.

### 3.2. Experimentally Obtained Temperature Profiles during the Printing Process

**Figure 5:**
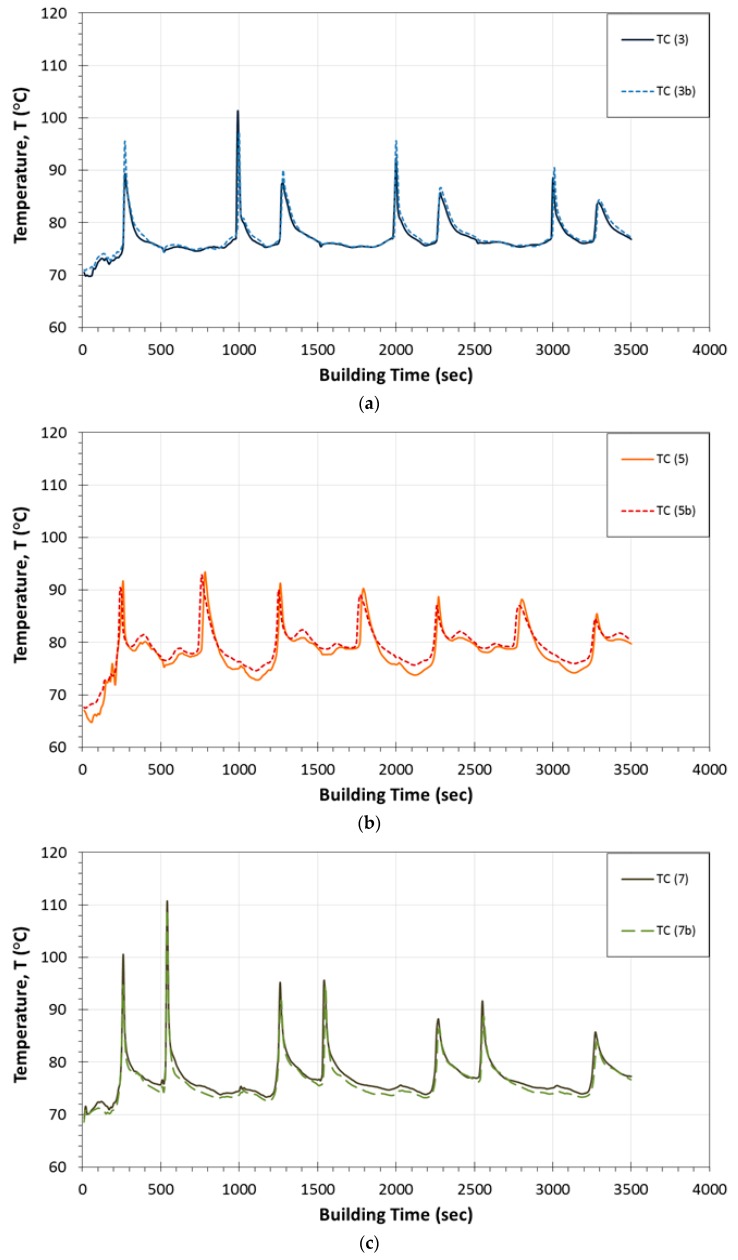
Comparison of temperature profiles measured during the printing process for embedding locations: (**a**) 3 and 3b; (**b**) 5 and 5b; (**c**) 7 and 7b.

In Figure 5a–c, the induced temperature profiles measured during the fabrication process are presented as a function of building time for points 3b, 5b and 7b located at the diagonal direction (+45°) of the plate specimen (dashed lines). In the same figure, these experimental results are compared to the temperature variations derived for locations 3, 5 and 7 during the fabrication process of the first, second and third type of the thin plate specimens (continuous lines). In Figure 5a,c, it is demonstrated that the only noticeable deviations between the data recordings obtained by the integrated thermocouples in locations 3;3b and 7;7b, for the three specimen types, are exhibited during the building process of the 7th layer and specifically at 260 s. This difference of 6 °C results due to the high temperature gradients generated at the corners of the printed structures. As far as Figure 5b is concerned, the temperature variations are observed to be almost identical in both types of the built samples. In conclusion, all temperature sensors positioned at the same locations of the different specimen types have led to repeatable recorded temperature profiles.

**Figure 6:**
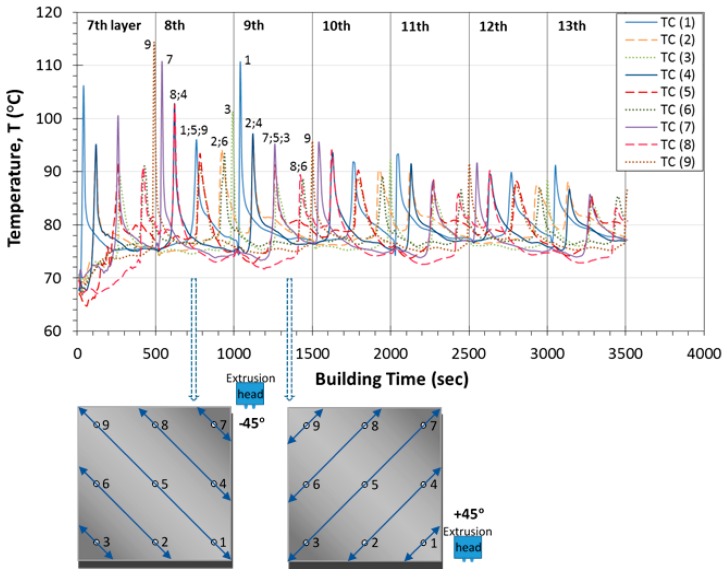
Mapping of temperature profiles generated within the FDM square plate specimens during the whole fabrication process.

The experimental data which map the temperature profiles during the printing process of the plate specimens are presented in Figure 6 as a function of the building time. The maximum temperature values (higher than 110 °C) are generated at locations 1, 7 and 9 that are quite close to the corners of the plates where the building process of each layer is initiated and completed. At the corners, the printer’s nozzle travels a much smaller distance to deposit the heated material. In addition, it moves at a faster speed compared to the middle sections of the plate specimens resulting to higher developed temperature gradients. Considerably high temperature values are recorded during the deposition of the first three layers, until 1040 s, while afterwards a smooth declining trend of all recorded profiles is present. Furthermore, as it is observed in Figure 6, the temperature gradients induced during the deposition of each layer (per 500 s) exhibit a decreasing trend until the passage of the nozzle exactly above the thermocouples located at positions 8;6 or positions 2;6 (±45°). After that time period, the measured temperature is rapidly increased again until the completion of the corresponding layer close to the location 3 or 9. This pattern is repeatable for all built layers except for the initially deposited one due to the peak value occurred at 250 s in location 7. The experimental results demonstrate also that the monitored peak temperatures at the right edges (1, 4 and 7) of the test samples are higher during the deposition of each layer (8th–13th) compared to the respective ones derived by the sensors incorporated within the left side’s edges (9, 6 and 3). During the printing process, the machine’s nozzle acts as a heat emission source. As a result, the previously described thermal behavior is greatly influenced by the nozzle’s building direction. This is also supported by the fact that the printer’s nozzle always starts the fabrication process from the right to the left side of the plate. From the previously presented results is concluded that the integration of temperature measuring sensors across the whole middle-plane of the plate structures can lead to an in situ and real-time mapping of the temperature profiles generated during the entire printing process. Such information regarding the temperature history of the plate specimens can assist with the investigation of the developed residual strains or stresses and consequently of the resulting warpage.

### 3.3. Simulation of Inter-Layer Temperature Distribution

**Figure 7:**
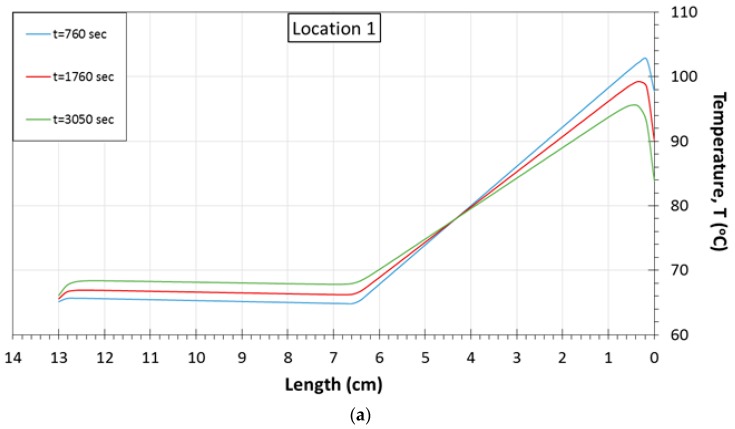

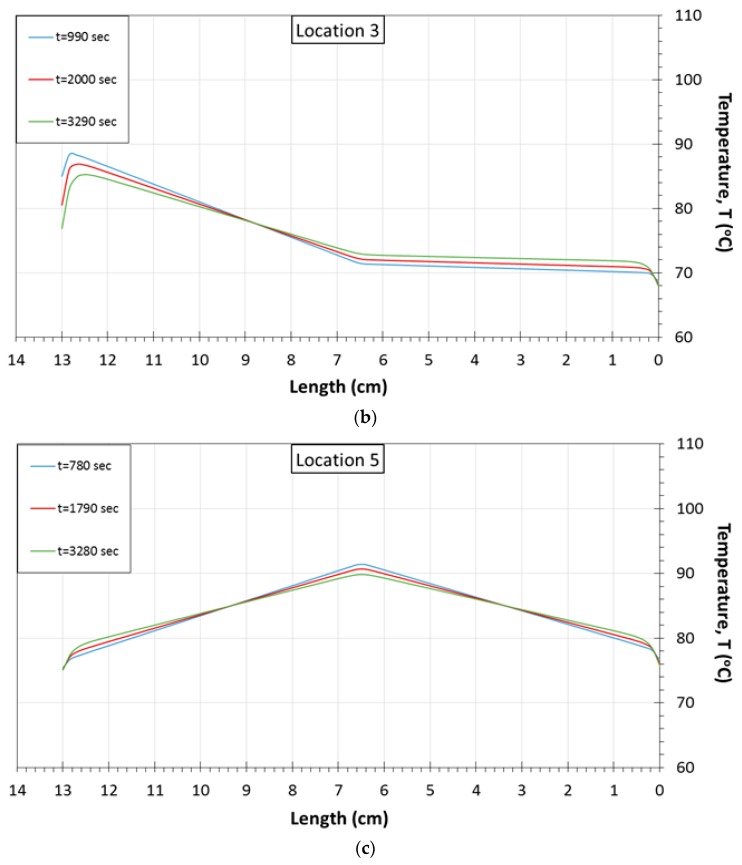
Temperature distribution across virtual lines of the 7th layer at different building times for thermocouple locations: (**a**) 1; (**b**) 3; (**c**) 5.

The diagrams shown in Figure 7a–c demonstrate the temperature distribution, as it is calculated by finite element analysis, across virtual horizontal lines of the middle-plane, at three different times of the fabrication process. Each virtual horizontal line has as reference zero-point the right side of the plate, passes through a set of three temperature measuring sensors each time, and finishes to the structure’s left edge. The graphs presented in this figure correspond to the numerical results obtained for thermocouple locations 1 and 3, which are close to the plates’ edges, as well as for the central location 5. As it is observed in Figure 7a,b, both graphs exhibit a similar trend at their calculated temperature distribution, which is reversed with respect to the length axis. As a result, in position 1 (Figure 7a), the maximum temperature value at 760 s of the building process is exhibited at 0.2 cm, whereas the corresponding one in location 3 (Figure 7b) is observed at 12.8 cm. Furthermore, for both graphs, a constant temperature plateau is exhibited at half length of the plate. In Figure 7a, the minimum and maximum temperatures as derived by the numerical model for the 7th layer at 760 s of the printing process are approximately 65 °C and 103 °C, respectively. In Figure 7b, the corresponding calculated ones vary from 70 °C to 88 °C. Therefore, it is noted that the temperature distribution induced at location 1 of the plate structure exhibit higher fluctuations compared to the simulation results derived for location 3. Furthermore, in both diagrams, it is observed that the deposition of the subsequent layers upon the surface of the 7th layer results in the reduction of the temperature peak value. This pattern is in accordance with the corresponding one obtained by the experimental results for locations 1 and 3 (see Figure 3a). On the other hand, the evident temperature plateau is increased with respect to the additionally built layers. As far as location 5 is concerned, in Figure 7c, the temperature peak values are shown exactly at the middle of the plate’s length. During the layerwise deposition process, the maximum calculated temperature value is also decreased. At a distance of 3.4 cm and 3.5 cm from the right and left edges of the structure, respectively, this thermal behavior is reversed resulting in the presence of higher temperatures at 3280 s. Finally, as the printing process is evolved, in all cases, the increase of the lower temperature values combined with the decrease of the higher ones indicate that the temperature distributions tend to become uniform across the simulated virtual lines of the 7th layer.

## 4. Conclusions

In the undertaken work, temperature-measuring sensors were integrated at various locations throughout the middle-plane of 3D printed polymer plates to investigate the temperature profiles generated during the building process. The experimental results were compared to the corresponding ones predicted by finite element analysis. It is shown that a good agreement was observed between the measured and the calculated temperature peak values, presenting a maximum difference of 5 °C at the lower edge side of the layerwise built plate. The deviations between the experimentally and numerically obtained values at the upper edge plate locations were due to the high temperature fluctuations induced during the fabrication process of the initially two built layers. The best possible correlation was achieved for those embedding positions located at the middle surface area of the plate specimens due to the display of uniform temperature profiles during the printing process. In all cases, the simulation based temperature peak values exhibited a decreasing tendency as a function of building time, eventually reaching up to a temperature plateau.

The experimental results demonstrate in a clear manner that considerably high temperature variations occurred during the printing process of the initially three deposited layers (until 1040 s). The maximum temperature values were recorded at locations close to the corners of the plate structures, where the initialization and completion of each layer’s building process were taking place. Additionally, the temperature peak values measured by the sensors incorporated within the right edges of the test samples were higher during the FDM process in comparison to those recorded at the left side’s edges. This thermal behavior is greatly influenced by the 3D printer’s nozzle building direction as well as its reference zero position within the machine’s chamber.

It is worth mentioning that the incorporation of temperature measuring sensors within various locations of the printed plate structures led to a comprehensive continuous in situ mapping of the temperature profiles generated during the building process. Such experimentally obtained data can provide the necessary information to proceed to the calculation of the corresponding developed strain fields as their magnitude strongly depends on the in-process induced temperature profiles.

## Figures and Tables

**Table 1:** Thermal properties of ABS.

Parameter	Values
Thermal conductivity, *k* (W/m·K)	0.177
Specific heat, *C_p_* (J/kg·K)	2.080
Density, *ρ* (kg/m^3^)	1.050

**Table 2:** Temperature peak values as recorded by two experimental runs for all thermocouple locations.

Built Layer	Experimental Run	TC1 (°C)	TC2 (°C)	TC3 (°C)	TC4 (°C)	TC5 (°C)	TC6 (°C)	TC7 (°C)	TC8 (°C)	TC9 (°C)
7th	1st	106.19	93.32	89.59	95.12	91.65	91.07	100.54	90.86	114.34
	2nd	109.85	95.77	92.47	97.09	95.55	92.06	‒	92.00	117.01
8th	1st	96.05	94.15	100.94	102.81	93.23	93.36	110.65	102.62	91.34
	2nd	99.69	91.28	98.76	100.67	94.55	92.28	‒	100.41	91.88
9th	1st	110.37	96.67	87.54	96.95	91.06	88.63	95.09	89.26	95.65
	2nd	111.87	94.09	88.25	99.51	92.48	92.14	‒	87.77	94.62
10th	1st	91.83	90.31	92.32	93.60	90.26	89.08	95.26	93.89	88.69
	2nd	91.37	88.68	91.97	94.90	91.17	90.56	‒	93.19	89.57
11th	1st	93.32	90.19	85.66	91.44	88.67	86.57	88.26	85.83	91.22
	2nd	93.51	88.99	85.21	94.70	88.79	88.55	‒	84.79	91.07
12th	1st	89.89	87.50	88.35	89.62	88.18	86.84	91.56	90.17	87.54
	2nd	89.36	86.60	87.53	93.02	88.47	87.70	‒	88.87	87.96
13th	1st	90.89	88.03	83.89	86.72	85.49	85.33	85.65	83.15	86.81
	2nd	90.48	86.74	83.82	88.53	86.05	86.10	‒	82.12	87.50
